# Switchable slow cellular conductances determine robustness and tunability of network states

**DOI:** 10.1371/journal.pcbi.1006125

**Published:** 2018-04-23

**Authors:** Guillaume Drion, Julie Dethier, Alessio Franci, Rodolphe Sepulchre

**Affiliations:** 1 Department of Electrical Engineering and Computer Science, University of Liege, Liege, Belgium; 2 National Autonomous University of Mexico, Science Faculty, Department of Mathematics, Coyoacán, D.F., México; 3 Department of Engineering, University of Cambridge, Cambridge, United Kingdom; SUNY Downstate Medical Center, UNITED STATES

## Abstract

Neuronal information processing is regulated by fast and localized fluctuations of brain states. Brain states reliably switch between distinct spatiotemporal signatures at a network scale even though they are composed of heterogeneous and variable rhythms at a cellular scale. We investigated the mechanisms of this network control in a conductance-based population model that reliably switches between active and oscillatory mean-fields. Robust control of the mean-field properties relies critically on a switchable negative intrinsic conductance at the cellular level. This conductance endows circuits with a shared cellular positive feedback that can switch population rhythms on and off at a cellular resolution. The switch is largely independent from other intrinsic neuronal properties, network size and synaptic connectivity. It is therefore compatible with the temporal variability and spatial heterogeneity induced by slower regulatory functions such as neuromodulation, synaptic plasticity and homeostasis. Strikingly, the required cellular mechanism is available in all cell types that possess T-type calcium channels but unavailable in computational models that neglect the slow kinetics of their activation.

## Author summary

Brain information processing involves electrophysiological signals at multiple temporal and spatial timescales, from the single neuron level to whole brain areas. A fast and local control of these signals by neurochemicals called neuromodulators is essential in complex tasks such as movement initiation and attentional focus. The neuromodulators act at the cellular scale to control signals that propagate at potentially much larger scales. The present paper highlights the critical role of a cellular switch of excitability for the fast and localized control of cellular and network states. By turning ON and OFF the cellular switch, neuromodulators can robustly switch large populations between distinct network states. We stress the importance of controlling the switch at a cellular level and independently of the connectivity to allow for tunable spatiotemporal signatures of the network states.

## Introduction

Neuronal processing is constantly shaped by fluctuations in population rhythmic activities, each defining distinctive brain states [1–4]. Neuromodulators organize the switch between different brain states [5,6], changing the way networks process neural signals [7,8]. Precise temporal and spatial control of brain states is required for changes associated with movement, attention, perception, motivation, or expectation [9–18]. Fast acting neurotransmitter pathways allow for the rapid kinetics required for fast network and signal processing states changes [17]. Rapid control of network states has been reported to affect spatial attention in cortical circuits [6,7], attention and arousal in the thalamus [7,18], and movement initiation in the subthalamic nucleus [14]. The most studied example is probably the thalamocortical circuitry. The thalamus acts as a plastic relay between sensory systems, different subcortical areas and the cerebral cortex, by gating and modulating neuronal signal flow under the modulatory effect of cortical feedback [19–22].

Experimentally, brain states are identified via specific spatiotemporal signatures of the mean-field electrical activity of large neuronal populations. Shifts in the rhythmic activity occur during transitions to slow-wave sleep and sleep spindles [1,7,8,23–27]. These shifts correlate with strong changes in the processing of afferent neuronal signals [8,17,28]. An extreme situation is when highly synchronized sleep oscillations develop into absence epilepsy, a behavioral state that can be viewed as a brain disconnection from the external world [29–31]. In the waking state as well, transient network state switches are observed and correlate with modulations of sensory-motor signals processing [16].

What are the mechanisms that enable fast and robust mean-field switches in heterogeneous neuronal populations that exhibit rhythms over a broad range of temporal and spatial scales, from single cells to networks?

At a cellular level, the rhythms are determined by specific balances of specific ionic currents. Specific synaptic connections determine specific circuit topologies that define new and different rhythms at a circuit scale. At a network level, the circuit topologies interconnect large and heterogeneous neuronal populations. Collectively, the populations shape a mean-field activity that defines yet another rhythm for the brain state. At every scale, the rhythms are continuously changing under the action of neuromodulators that modulate cellular and synaptic conductances over time. Neuromodulatory systems act at a cellular scale but their broad projections can simultaneously affect large populations [6,7,17,27]. Global neuromodulators control the switch of brain states [16,32–34]. The question we wish to investigate in the paper is how this global control can cope with—and in fact benefit from—the heterogeneity and variability of rhythms at a cellular scale.

A similar question has received considerable attention over the last two decades in the study of neuromodulation of small rhythmic circuits controlling the pyloric and gastric mill rhythms of the crab [35–38]. This work has elucidated to a great extent how heterogeneity and variability at the cellular scale is not only compatible with homogeneity and stability at the circuit scale but in fact an essential source of robustness and tunability in circuit rhythms. The present paper is inspired by this line of work: we developed a simple conductance-based computational model to investigate how heterogeneity and variability at the cellular and circuit scales contribute to the robust control and tunability of brain states.

Previous computational models of brain states have focused on the role of connectivity changes in network rhythm modulation [39–41]. To account for fast fluctuations, our model instead studies network switches that do not require changes in network connectivity. We propose that the mean-field switch results from a cellular switch that is shared by a sufficient fraction of the population. This mechanism is largely independent of the network topology and the network connectivity is always assumed to be *weak*. This is what allows for rhythmic heterogeneity within the population. The cellular switches only control a discrete transition between two distinct modes of excitability, classically referred to as tonic firing and bursting [42]. The weak connectivity makes this discrete transition compatible with a continuum tuning of each discrete state. The homogeneous control of brain states at a network level only relies on the shared cellular switch. It is compatible with heterogeneous and variable rhythms at a cellular and circuit scales.

Our paper aims at showing the computational and physiological relevance of this novel mechanism both for network studies and cellular physiology. Regarding the network computations, we show that heterogeneity and variability at the cellular and circuit scale promote robustness and tunability at the network scale. The cellular *switch* decouples the control of network states, which is fast and global, from the *tuning* of the spatiotemporal rhythm, which is ensured by modulation of intrinsic and synaptic properties at slower temporal and finer spatial scales. The decoupling of tasks between *switching* and *tuning* allows for a fine regulation of both the oscillatory and active states of the network. This tuning of states has essential functional relevance, such as modulating the transmission properties of the network in its active state.

At a physiological level, our results stress the role at a network level of specific ionic mechanisms that have long been studied in single cell neurophysiology but are often neglected in network studies for the sake of numerical or mathematical tractability. The cellular switch of our model entirely relies on a tunable slow negative conductance. The switching role of this slow conductance has been studied in a series of recent papers by the authors. It regulates the cellular modulation of excitability types [43–46] and in particular the transition between tonic firing and bursting [47,48]. It is critical for the robustness and tunability of cellular bursting [42]. And it is critical to the robustness of rhythmic circuits such as half-center oscillators [49]. In the continuation of this research, the present paper shows how slow negative cellular conductances contribute to network neuromodulation, highlighting the physiological importance of a specific cellular mechanism at a network level.

## Results

### Robust and tunable network states made of heterogeneous cellular and circuit states

Our computational model reproduces a generic example of network switch between an active and an oscillatory state in a population of neurons. At the cellular level, we used a conductance-based model that includes the typical fast and slow ionic currents of bursting cells. Each cell can be robustly modulated by a hyperpolarizing input between two distinct modes of excitability: a *fast depolarized* mode, prone to spiking and tonic firing, and a *slow hyperpolarized* mode, prone to bursting.

At the network level, we included AMPA, GABA_A_ and GABA_B_ connections to model the asymmetric coupling between a subpopulation of excitatory (E) cells and a subpopulation of inhibitory (I) cells. This topology is typical of brain areas involved in state regulation, such as e.g. the thalamus [7], the cortex [2], and the subthalamic nucleus/globus pallidus [39]. Our model neglects intra-population connectivity, which maximizes the heterogeneity of cellular rhythms within each subpopulation. In contrast, it assumes all-to-all connectivity between the two populations, but with weak and randomly distributed synaptic weights (see methods for details). We explored rhythmic properties of the network that allow for a broad heterogeneity of both intrinsic and synaptic maximal conductance parameters. This parametric heterogeneity generates a broad range of rhythms at cellular and circuit levels.

**Fig 1:**
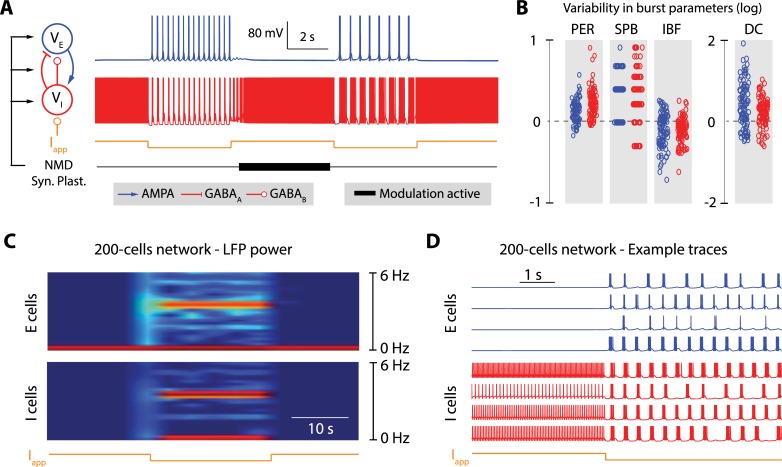
A robust network switch that is compatible with global neuromodulation, synaptic plasticity and homeostasis. **A.** Variations of the firing pattern of two interconnected neurons (one excitatory neuron, in blue, and one inhibitory neuron, in red) under the control of an external current (I_app_, in orange), slow neuromodulators (NMD, in black), and synaptic plasticity (Syn. Plast., in black). The tick, black trace depicts periods during which the slow neuromodulator and synaptic plasticity are active. They are modeled as random changes in ion channel and receptor maximal conductances. The excitatory neuron is connected to the inhibitory neuron via AMPA synapses, and the inhibitory neuron is connected to the excitatory neuron via GABA_A_ and GABA_B_ synapses. The external current, which transiently hyperpolarizes the inhibitory neuron, switches the rhythm of the circuit. The fast switch is robust to the rhythmic variability induced by slow neuromodulators and synaptic plasticity (compare the rhythm generated by the fast switch before and after the action of NMD and Syn. Plast.). **B**. Quantification of the variability of rhythms that can be generated by the action of slow neuromodulators and synaptic plasticity without disrupting the fast switch. The figure plots the difference in bursting period (PER), number of spike per bursts (SPB), intraburst frequency (IBF) and burst duty cycle (DC) of the fast switch-induced rhythm before and after the application of 100 different tones of slow neuromodulation and Syn. Plast. (log(parameter_after/parameter_before)) **C**. Spectrogram of the local field potentials (LFP’s) of excitatory neuron (top) and inhibitory neuron populations in a 200-cell network (100 excitatory cells fully connected to 100 inhibitory cells with random synaptic weights taken within a fixed range). The orange trace at the bottom depicts the period during which the external hyperpolarizing current is applied to the inhibitory neurons. The hyperpolarization is shown to transiently switch the mean field rhythm of the population, which is shown by the appearance of a transient high power band in the spectrogram. **D.** Example traces of single neuron activity in the excitatory (in blue) and inhibitory subpopulations (in red) for the network switch shown in **C**. The orange trace at the bottom depicts the period during which the external hyperpolarizing current is applied to the inhibitory neurons.

The circuit and network switches are illustrated in Fig 1. They are controlled by transient hyperpolarizations of the inhibitory neurons, which for instance mimic activation of GABA_B receptors. At the cellular level, the hyperpolarization induces a switch from the fast excitability mode to the slow excitability mode. The cellular switch in turn induces a switch at the circuit level because the slow mode of excitability induces rebound mechanisms between the excitatory and inhibitory cell. Fig 1A and Fig 1B illustrate the robustness and modulation capabilities of the rhythms in isolated E-I circuits of two cells. Fig 1A shows that a transient hyperpolarization can reliably induce a switch from asynchronous spiking to synchronous bursting, and that this switch is robust to changes in neuron intrinsic and synaptic properties induced by a persistent, global neuromodulation ("NMD") and synaptic plasticity ("Syn. Plast."). Thanks to the robustness of the cellular switch to variability, the circuit rhythm is robustly maintained over broad intrinsic and synaptic parameter ranges. As a result, a persistent neuromodulator affecting both intrinsic and synaptic parameters can modulate the cellular and circuit rhythms between successive occurrences of the transient hyperpolarization (Fig 1A and 1B).

At the network level, the mean field of the population defines a network switch between an *oscillatory* state, corresponding to the slow mode of cellular excitability, and an *active* state, corresponding to the fast mode of cellular excitability. The network switch is robust to temporal variability and spatial heterogeneity of the population because the cellular switch exists over a broad range of intrinsic and synaptic parameters. This robustness makes the external control of the network largely independent of the network size and connectivity. Fig 1C and 1D illustrate that property in a heterogeneous network of 200 cells. The local field potential (LFP) activity illustrates that the transient hyperpolarization can turn on and off the mean-field rhythmic activity (defined by a marked high power LFP frequency band) of the entire controlled population. Fig 1D further shows that this population control is compatible with heterogeneous rhythms at a cellular and circuit resolution. In other words, reliable control of the network state is compatible with rhythmic heterogeneity and variability at a cellular resolution. The mean-field network rhythm does not result from the synchronization of the cellular or circuit rhythms. Instead, the rhythmic network state arises from a shared cellular switch that is robust to the variability of tunes at a cellular resolution.

### Switching the network state of a heterogeneous population critically relies on a cellular property

Our computational model exhibits a robust mean-field switch at the network level in spite of heterogeneity and variability at the cellular level. Such a property is not granted in a computational model that depends on thousands of uncertain and variable parameters. In our computational model, it critically relies on a switchable *slow negative conductance* at a cellular level.

**Fig 2:**
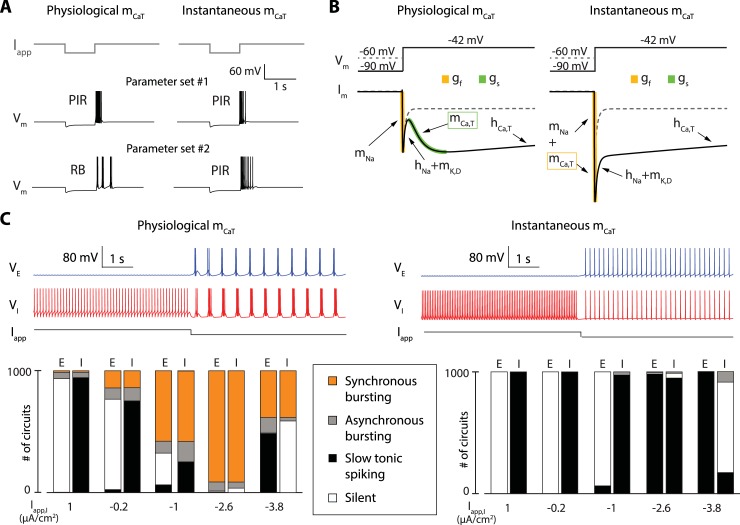
The cellular switch requires the kinetics of T-type calcium activation to be slow; it is lost when the activation is modeled as instantaneous. **A**. Response of the model neuron to the application of transient hyperpolarization for two different parameter sets in two models that only differ in the activation kinetics of T-type calcium channels, which is either physiologically slow (left) or instantaneous (right). For the first parameter set (middle traces), a release of the hyperpolarization induces the generation of a transient spiking period in both models, a property called post-inhibitory rebound (PIR). This observation shows that PIR is robust to T-type calcium channel activation kinetics. For the second parameter set (bottom traces), a release of the hyperpolarization induces the generation of a transient bursting period in the model having slow T-type calcium channel activation kinetics (left), a property called rebound bursting (RB), whereas it induces a PIR in the model having instantaneous T-type calcium channel activation kinetics (right). This observation shows that, contrary to PIR, RB is sensitive to T-type calcium channel activation kinetics. **B**. Voltage-clamp experiments in two single neuron models that only differ in the activation kinetics of T-type calcium channels, which is either physiologically slow (left) or instantaneous (right), all other parameters being identical. The top traces show the two voltage steps applied to the neurons. These steps only differ in the initial, holding potential, which is either -60 mV (dashed grey trace) or -90 mV (full black trace). The bottom traces show the ionic currents recorded over time during the application of either voltage steps (the dashed grey traces are the current corresponding to the -60 mV holding potential, the full black traces are the current corresponding to the -90 mV holding potential). The responses of both models to the step starting at -60 mV are the same (T-type calcium channels are inactivated, and all other parameters are identical between the two models). The responses of both models to the step starting at -90 mV are very different. The model with physiologically slow T-type calcium channel activation kinetics show two phases of increasing inward current, a fast one (in orange) and a slow one (in green). The model with instantaneous T-type calcium channel activation kinetics show only one amplified fast phase of increasing inward current (in orange). Both current traces however reach the same current level at steady-state, showing that the difference between the two models is of dynamical nature. **C.** Comparison of the switching capabilities in 2-cell circuits with random intrinsic and synaptic conductances using neuron models with physiologically slow T-type calcium channel activation kinetics (left) or instantaneous T-type calcium channels activation kinetics (right). The top traces show examples of neuronal activity before and after the application of a hyperpolarizing current onto the inhibitory neuron (the excitatory neuron is depicted in blue, and the inhibitory neuron is depicted in red). The bottom bar graphs quantify the activity of 1000 simulated random circuits under the application of 5 different applied currents. Cells are either silent (white), spiking slowly (black), bursting asynchronously (grey) or involved in a synchronous bursting rhythm (orange). With slow activation of T-type calcium channels, most of 1000 simulated random circuits switch from fast to slow rhythms under hyperpolarization (left). None of them was found to switch when the activation is instantaneous (right).

The slow negative conductance of a neuron determines its slow excitability in the same way as the fast negative conductance determines its fast excitability. In the same way as sodium channel activation enables the fast switch from rest to spike, a slow negative conductance enables the slow switch from rest to burst. Ion channels that can contribute to the slow negative conductance of a neuron are called *slow regenerative* [45,46,48]. A channel is slow regenerative if it activates an inward current or inactivates an outward current in a time scale that is slow relative to the fast time scale of sodium activation. In our model, only the T-type calcium channels are slow regenerative. Their *activatio*n is *slow* relative to sodium channel activation [50]. Moreover, because of their inactivating, low threshold nature, T-type calcium channels equip the neuron with a slow negative conductance that is *switchable* by an external current: it is turned on by hyperpolarization and turned off by depolarization. This switching mechanism is distinct from the classical rebound mechanism (called post-inhibitory rebound, or PIR) associated to T-type calcium channels and other transient inward currents such as hyperpolarization-activation cation currents [7,49,51,52]. It endows the cell with slow excitability. The experimental manifestation of this slow excitability notably includes rebound bursting (RB) (Fig 2A, left) and hyperpolarization-induced bursting (HIB) (S1 Fig). Such behaviors have been widely observed experimentally [7,53–58].

We have studied in detail in [42] why a slow negative conductance is critical to a cellular activity that allows for a robust and controlled switch between fast and slow tunable rhythms, and how this mechanism relates to bursting models of the literature, such as square wave bursting or parabolic bursting. A similar observation applies to the two-cell excitatory-inhibitory circuit of Fig 1A. The circuit rhythm results from a well-known rebound mechanism [7,51], but it does do so only when the slow negative conductance is turned on. In order to assess the specific role of the slow negative conductance, we proceeded with the same protocol as in [42]: we compared a nominal model in which the activation kinetics of T-type calcium channels is physiologically *slow*, (about ten time slower than the activation kinetics of sodium channels) to a perturbed model in which the activation kinetics of T-type calcium channels is *instantaneous*, that is, undifferentiated from the activation kinetics of sodium channels. Instantaneous activation of calcium channels is a frequent modeling assumption (see Discussion). In both the nominal and perturbed models, T-type calcium channels provide an inactivating inward current necessary for the rebound mechanism (Fig 2A). However, the slow excitability mode is lost in the perturbed model because both sodium and calcium channels only contribute to the fast negative conductance. This change has a clear signature in a voltage-clamp experiment (Fig 2B). In the nominal model, a voltage step from hyperpolarized potential (-90mV) deinactivates T-type calcium channels, which results in two temporally distinct phases of negative conductance: a fast one (depicted in yellow in Fig 2B, left) and a slow one (depicted in green in Fig 2B, left). The specific signature of the slow negative conductance is not present in the perturbed model. Instead, the two negative conductances add up in the fast time scale. The chosen perturbation has the advantage that it does not affect the model properties at equilibrium: the nominal and perturbed models have the same I/V curve and the same balance of currents at steady-state. Fig 2C illustrates that the slow kinetics of calcium channel activation is essential to make the switch of a 2-cell E-I circuit robust to parameter variability. A thousand 2-cell networks were simulated by randomly generating 1000 different parameter sets for maximal intrinsic and synaptic conductances (see Methods). Fig 2C, left shows that the circuit switch occurs in the vast majority of parameter configurations when T-type calcium channel activation is physiological (>90% for I_app_ = -2.6μA/cm^2^). This is the robustness that allows for variability, modulation (Fig 1A and 1B), and heterogeneity (Fig 1C and 1D) at the cellular resolution.

In contrast, robustness and tuning properties of the cellular and circuit rhythms are totally lost when the calcium activation is fast (Fig 2C, right). The rhythm of the E-I circuit is extremely fragile to the loss of slow regenerative ion channels. It requires a very precise tuning of both the ionic (intrinsic) and synaptic (extrinsic) parameters. No circuit rhythmic activity could be found out of the 1000 random parameter sets simulated in this configuration. In the absence of slow regenerativity, the switch of the circuit cannot be decoupled from the rhythm of the circuit: both exist only for very specific parameter sets.

### Heterogeneity at cellular resolution promotes robustness and tunability of network states in larger populations

**Fig 3:**
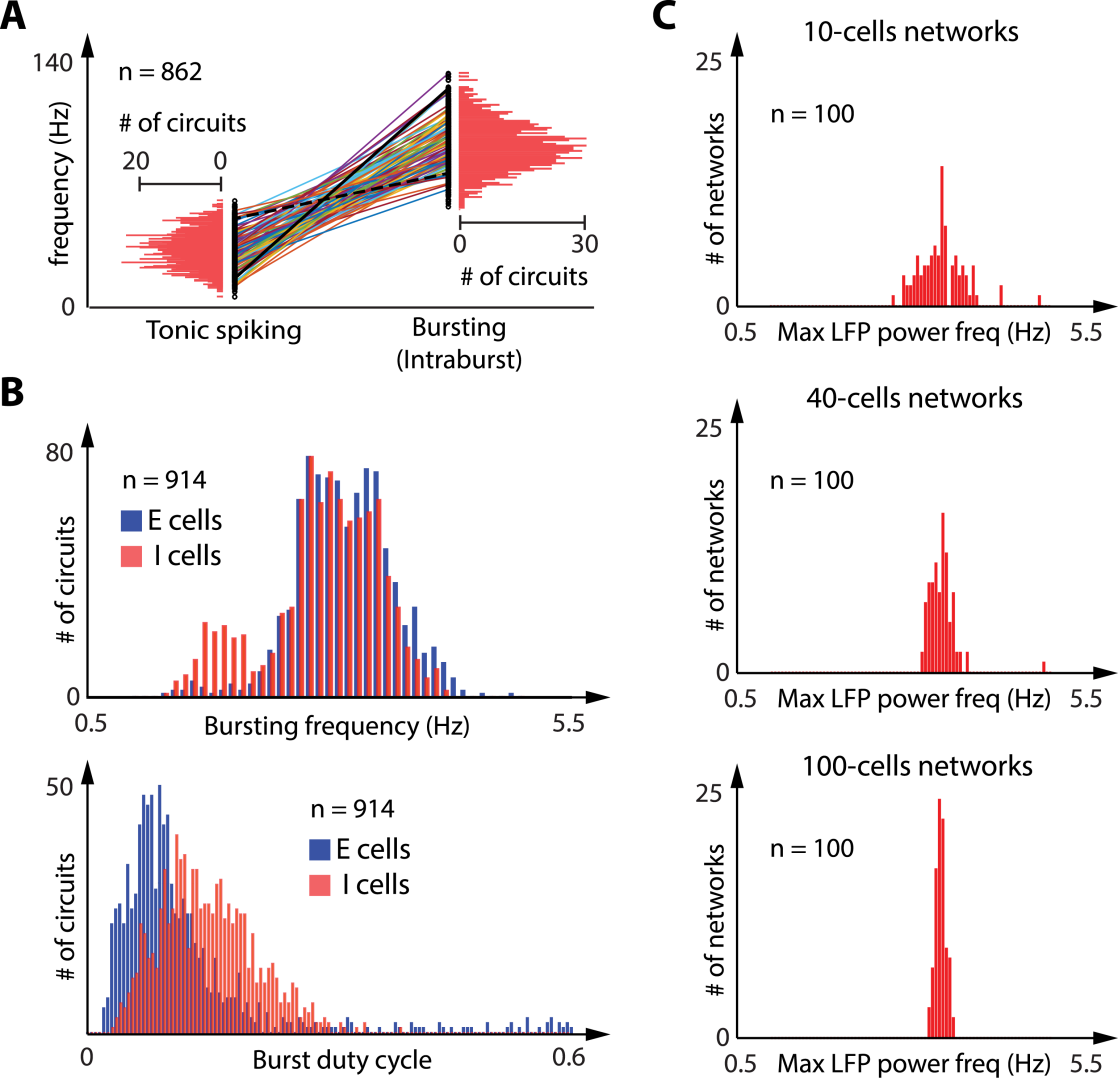
The robustness of the cellular switch to intrinsic and synaptic variability allows for a large tuning of rhythmic properties. **A**: Comparison of the tonic spiking frequency before external hyperpolarization and the intraburst frequency during external hyperpolarization in the 862 random 2-cell circuits (out 1000 random 2-cell simulated circuits) that showed a hyperpolarization-induced switch from tonic spiking to synchronized bursting (values are shown for the inhibitory neurons, the excitatory neurons being mostly silent during the depolarization period). Both values are given for every inhibitory cells (black dots), and a line links values for the same cells. The horizontal red bars quantify the occurrence of cells within each frequency range. Both tonic firing and intraburst frequencies show large variability within the population, but intraburst frequency is consistently higher than tonic firing frequency in a given neuron. **B:** Comparison of the bursting frequency (top) and the burst duty cycle (bottom) in the 914 random 2-cell circuits (out 1000 random 2-cell simulated circuits) that exhibited a synchronized bursting rhythm during hyperpolarization. Vertical bars quantify the occurrence of excitatory cells (in blue) and inhibitory cells (in red) within each bursting frequency range (top) or within each duty cycle range (bottom). Bursting frequency and bursting duty cycle show large variability within the population, showing that a same circuit switch can produce very different circuit rhythms. **C**: Distribution of maximum LFP power frequency during hyperpolarization in 100 random, highly heterogeneous 10-cells networks (top), 40-cells network (middle) and 100-cells networks (bottom). The red vertical bars show the occurrence of networks within each frequency range. The mean field spectral power shows large variability within the network population for small networks, but the spread of the mean field spectral power gradually shrinks as the population size increases.

Fig 3A and 3B illustrate the continuous tunability of rhythms at the circuit level in the two discrete excitability modes. Fig 3A shows that the frequency of tonic firing and the intraburst frequency of the bursting mode can be modulated over a broad range (several fold). All cells verify the physiological property that the intraburst frequency is significantly higher than the tonic firing frequency, a key feature of bursting signaling and a physiological signature of hyperpolarization-induced bursting [7,53–55]. Fig 3B shows that two properties of the slow rhythm (bursting frequency, top, and burst duty cycle, bottom) can also be modulated over a broad range (more than 5 fold) without affecting the hyperpolarization-induced switch. Fig 3C illustrates the mean-field property of our network: in spite of the heterogeneity of rhythms at a cellular and circuit scales, the variability of the rhythms in the population progressively shrinks as the network size increases and eventually vanishes for very large neuronal populations (mean-field limit). The existence of two discrete mean-fields is a consequence of the cellular switch. Heterogeneity and variability in the population contribute to the tunability of the two discrete mean-fields.

### A spatio-temporal localized control of network state

Because of its cellular nature, the network switch described in this paper allows for precise spatio-temporal control of the population: its temporal resolution is only limited by the kinetics of the slow regenerative channels; its spatial resolution is only limited by the spatial resolution of receptors to neuromodulatory inputs. Such a mechanism enables spatiotemporal control of a network state at multiple resolutions.

**Fig 4:**
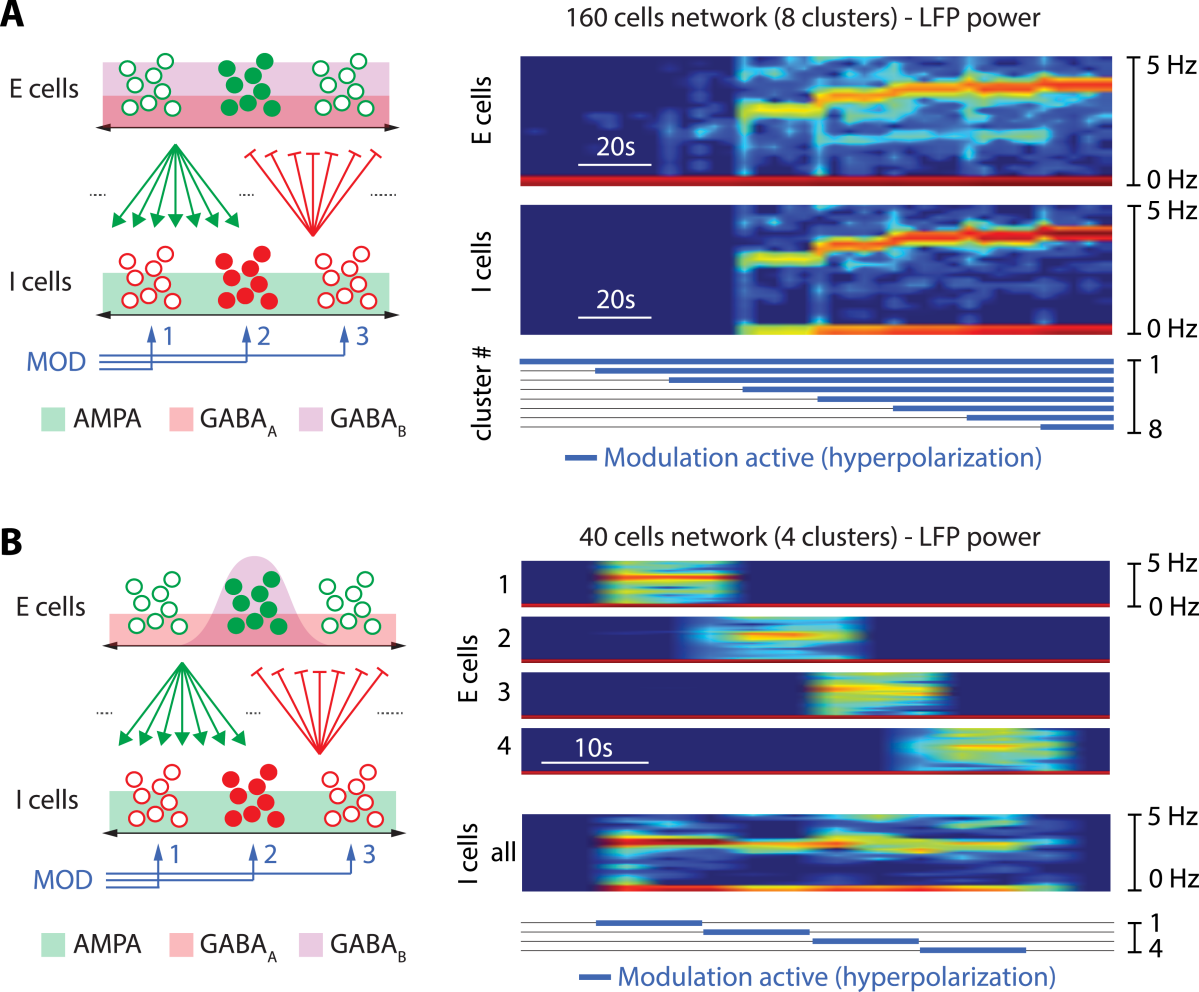
The cellular nature of the switch allows for localized spatiotemporal control of the network state. **A, left**. Sketch of the spatial network connectivity and clustering in modulatory pathways. Excitatory cells are sketched by (full and empty) green dots, inhibitory cells by (full and empty) red dots, excitatory synapses by green arrows and inhibitory synapses by red lines with perpendicular bars at their tip (excitatory and inhibitory cells are connected in a all to all fashion). The horizontal, double black arrows represent the spatial dimension, and the green, red and purple background the spatial spread of AMPA, GABA_A_ and GABA_B_ synapses coming from the neurons represented by full dots, respectively (synapses are spread evenly in this case). The blue arrows represent different modulatory pathways (3 pathways are represented: MOD 1, 2 and 3). Each modulatory pathway only affects a subpopulation of the inhibitory cells, represented by grouped sets of red dots. **A, right**. Spectrogram of the local field potentials (LFP’s) of excitatory neuron (top) and inhibitory neuron populations (bottom) in a 160-cell network (80 excitatory cells fully connected to 80 inhibitory cells with random synaptic weights taken within a fixed range) separated in 8 clusters, each modulated by a specific neuromodulatory pathway. The tick, blue traces at the bottom depict the period during which the hyperpolarization is active for each neuromodulatory pathway (from 1 to 8). Spatial localization of modulatory inputs affects the temporal tuning of mean-frequency and amplitude of the LFP spectral power. **B, left:** Same as **A, left**, expect for the fact that, here, GABA_B_ synapses are clustered by the addition of a spatial Gaussian decay in the synaptic strength (the GABA_B_ synapse is strong for neighboring neurons but weak for distant neurons). AMPA and GABA_A_ synapses are still spread evenly. **B, right**. Spectrogram of the local field potentials (LFP’s) of 4 clusters of excitatory neurons (top) and the inhibitory neuron populations in a 40-cell network (20 excitatory cells fully connected to 20 inhibitory cells with random synaptic weights taken within a fixed range and spatial clustering in GABA_B_ synapses). Both population are separated in 4 clusters, each modulated by a specific neuromodulatory pathway. The tick, blue traces at the bottom depict the period during which the hyperpolarization is active for each neuromodulatory pathway (from 1 to 4). Spatial localization of modulatory inputs and GABA_B_ connectivity affects the spatial tuning of the network state. Spatial localization of the GABA_B_ connections only allows for spatial control of the network state, even though AMPA and GABA_A_ connections are all-to-all.

Fig 4 illustrates two simple forms of spatiotemporal control in our model. Fig 4A shows how the network state of a 160-cell population is affected by the number of active hyperpolarizing neuromodulatory pathways. For the sake of the illustration, the neuromodulatory input is equally divided into 8 pathways. A global network rhythm is induced through the hyperpolarization of a sufficient subpopulation of I-cells (4 pathways out of 8 in the chosen illustration). Both the rhythmic spectral power and the frequency distribution are modulated by the number of active pathways. This example shows that the temporal properties of a network rhythm can be modulated by spatially varying neuromodulatory inputs, even in the absence of changes in either intrinsic or synaptic parameters of the network.

Fig 4B illustrates the spatial modulation of a network state in a fully connected population. In the proposed excitatory-inhibitory topology, a spatial clustering in GABA_B_R only is sufficient to create clusters in the network switch of the excitatory population (Fig 4B, left). When one of the neuromodulatory pathways is activated, it only affects the E-cells that have sufficiently strong GABA_B_ connections with the modulated inhibitory subpopulation. As a result, the LFPs of the excitatory subpopulations are orchestrated individually and the temporal rhythm is modulated along the spatial axis: each spatially localized neuromodulatory pathway can switch ON and OFF a corresponding rhythm (Fig 4B, right). Each rhythm has a specific spectral power signature, and the spatial organization of the network state is controlled at the spatial resolution of the neuromodulation.

In contrast to the excitatory population, the rhythmic activity of the inhibitory population is spatially uniform in our illustration. This is because the AMPA connections from the excitatory to the inhibitory population are not clustered. As a result, the spatiotemporal organization of the network states is different in each subpopulation.

The spatiotemporal control of the network entirely rests on decoupling the switching control from the tuning control. The switching is always at a cellular resolution. The tuning is at any temporal scale slower than the cellular rhythm and at any spatial scale between the cellular and network level. Because it controls the switch, a slow negative conductance at the cellular level is again critical to the spatiotemporal controllability of the network.

### Control of the network transfer properties by modulation of the active state

**Fig 5:**
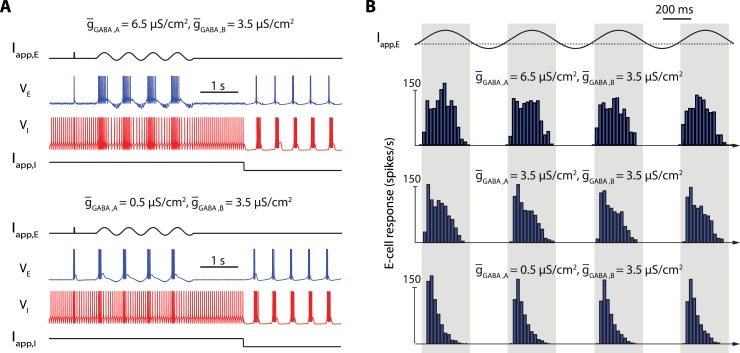
The network switch is robust to a modulation of the transmission properties in the active network sate. **A**: Response of excitatory cells to a pulse of excitatory current and a sinusoidal applied current for a high GABAR_A_/GABAR_B_ ratio (top) and a low GABAR_A_/GABAR_B_ ratio (bottom) within a 40-cells network. For each GABAR_A_/GABAR_B_ ratio, the top, black trace show the current applied to the excitatory neurons over time, the blue trace shows the activity of one targeted excitatory neuron over time, the red trace shows the activity of an inhibitory neuron over time, and the bottom, black trace show the current applied to all inhibitory neurons of the population over time. **B**: Mean excitatory cells response (in spikes per seconds) to a sinusoidal applied current for a high (top), intermediate (center) and low GABAR_A_/GABAR_B_ ratio (bottom). Blue vertical bars show the mean spiking rate for each period of time. A change in the GABAR_A_/GABAR_B_ ratio strongly affects the relay properties of the excitatory cells in active state without disrupting the network switch, and bursting in relay cells alone does not trigger the network switch, making this firing pattern compatible with the network active state.

Our analysis so far has focused on the tuning of the slow rhythms in the oscillatory network state. Fig 5 illustrates how the model also accounts for robust tuning of the active state, which is critical to modulate the transmission properties of a population.

The figure shows how E-cells process impulses and sinusoidal inputs in two different configurations. The only difference between the two configurations is the ratio between GABA_A_ and GABA_B_ synaptic connection strength. This is consistent with a physiological regulation through synaptic plasticity [56,57]. The switch control of the network is insensitive to the synaptic ratio but the transmission properties of the E-cells are markedly affected (Fig 5A). A high ratio enhances a linear-like response of E-cells: a short pulse of excitatory current triggers a spike, and a sinusoidal input entrains a phase-locked train of bursts (Fig 5A, top). In contrast, a low ratio enhances a detector-like response: a short pulse of excitatory current triggers a burst of spikes, and a sinusoidal input triggers burst of spikes whose frequency is maximum at the onset of the rising phase of the input signal (Fig 5A, bottom). This modulation of transmission properties is further quantified in Fig 5B, which illustrates the average response of a neuronal population to a sinusoidal input for three different synaptic ratios. For high ratio, the peak response is reached at the peak amplitude of the input signal. For low ratio, the peak response is reached when the input signal crosses a threshold from below. Such a modulation of the transmission mode is reminiscent of physiological observations in thalamocortical loops [7,8].

We emphasize that the modulation of the active state illustrated in Fig 5 is once again critically dependent on a slow negative conductance at the cellular level. The ration between GABA_A_R and GABA_B_R primarily regulates the membrane polarization. When GABA_A_R connections dominate, the inhibitory drive from the I-cells maintains E-cells close to GABA_A_R reversal potential, i.e. chloride reversal potential (set to -70mV in our model). At this potential, T-type calcium channels are inactivated, and the slow negative conductance is turned off: the E-cells exhibit the physiological signatures of the fast excitability mode: spike excitability and tonic firing [45,47,48]. In contrast, when GABA_B_R connections dominate, the inhibitory drive maintains E-cells close to GABA_B_R reversal potential, i.e. potassium reversal potential (set to -85mV in this model). At this potential, T-type calcium channels are deinactivated, and the slow negative conductance is turned on: the E-cells exhibit the physiological signatures of slow excitability: burst excitability and endogenous bursting [45,47,48].

## Discussion

### Separating the control of switching and tuning

In an effort to model the spatiotemporal organization of brain states, we proposed a simple neuronal network architecture that exploits modulation and heterogeneity of rhythms at a cellular resolution to tune the spatiotemporal signature of large rhythmic populations. At the core of our model lies a separation between switching mechanisms and tuning mechanisms. The mechanism of the switch is simple. It involves a single cellular property, occurs at a single temporal scale, and it is uniformly shared in the population. In contrast, the tuning mechanisms are multiple. They involve both cellular and synaptic properties, occur at many temporal and spatial scales, and can be highly heterogeneous in the population. A transition between two discrete states exists at every scale, from cells to networks, because of the uniform cellular switch. In contrast, the spatiotemporal signatures of each discrete state can be continuously tuned across the entire population, shaping robust and tunable network states. The central contribution of our model is to show that the cellular switch is essential to shape the network properties. It is necessary to the robustness of the network switch and enables the tunability of the network states by the remaining intrinsic and extrinsic conductances.

### A specific role for T-type calcium channels

The network control described in this paper only rests on two specific features: a cellular property to control the intrinsic slow negative conductance, provided by T-type calcium channels in our model, and a network topology that reciprocally interconnects an excitatory subpopulation and an inhibitory subpopulation. Those two properties are widely shared among a variety of circuits that exhibit fast control of network rhythms [7,8,13–19,30,53–55,58–67]. The canonical example is the thalamus, where both the role of rebound rhythms between (excitatory) thalamocortical neurons and (inhibitory) reticular neurons and the importance of T-type calcium channels have long been recognized in controlling network oscillatory states associated to sleep and attention [7,8,30,53–55,58–62,65]. Basal ganglia provide another example where the control of beta oscillations has been linked to rebound rhythms between (excitatory) subthalamic neurons and (inhibitory) external globus pallidus (GPe) neurons. A large amount of recent experimental evidence also demonstrates the importance of T-type calcium channels in the modulation of those rhythms. At the cellular level, experimental evidence shows that both STN and GPe neurons possess the ionic currents to undergo an excitability switch [53,54,64]. At the network level, oscillations have been recorded in the STN-GPe network in vitro and in vivo [13,14,39]. Fluctuations of the network state, and more specifically in the coherence and strength of beta oscillations have been linked to voluntary movement initiation, both in animals [68,69] and in humans [14,68,70–73]. Experimental studies show a prospective increase in beta synchrony prior to voluntary movements [74] and an event-related desynchronization in the beta band during movement [70, 72]. Initiation of voluntary movements is also linked to an increase in dopamine and, in particular, to a transient increase in the activity of nigrostriatal circuits (phasic dopamine release) [75,76]. This dopamine transient increase triggers the decrease of the beta-band activity coherence and power [77]. Those observations are consistent with the predictions of our model under a transient modulatory input. Fluctuating brain states have also been described in the cortex. Models of the layer V of the cortex involved in vision include an excitatory-inhibitory network and T-type calcium currents [78]. In this brain region, oscillatory activity in the alpha band (8–12 hertz) gates incoming signals by inhibiting task-irrelevant regions, thus routing signals to task-relevant regions [79,80]: alpha oscillations provide a functional inhibition and reduce the processing capabilities.

### A cellular switching mechanism distinct from rebound properties

At a cellular level, the excitability switch modeled in this paper is responsible for rebound bursting and hyperpolarization-induced bursting, two mechanisms that have been widely observed in experiments [7,53–55,58–60]. We stress that the excitability switch is distinct from the extensively studied post-inhibitory rebound (PIR) [7,49,51,52]. For instance, T-type calcium channels contribute to the switch mechanism through their slow *activation*, which is an intrinsic source of slow *negative* conductance, while they contribute to the post-inhibitory rebound through their *inactivation*, which is an intrinsic source of *positive* conductance. Other channels, such as HCN channels, only contribute to the rebound but do not contribute to the switch. The importance of T-type calcium channels has long been emphasized for their contribution to rebound mechanisms, both in central pattern generators and in mammalian brain rhythms [7,49,51,52]. The novelty of our model in that regard is to stress the importance of T-type calcium channels for their contribution to the *switch* in network state control. In the absence of the switch, rebound mechanisms alone do not suffice for network control of robust and tunable network rhythms. In the absence of slow regenerativity, a rebound rhythm in an excitatory-inhibitory network requires a specific resonance between the PIR and the kinetics of synaptic connections [41]. In this case, the circuit rhythm is fragile to changes in neuron intrinsic properties and synaptic connectivity. This fragility severely restricts the heterogeneity of rhythms in the population.

A particular manifestation of the distinction between excitability switch and rebound property is provided in Fig 5. In this figure, excitatory cells exhibit bursts both in the active and oscillatory states of the network. However, they participate in a rebound mechanism only in the oscillatory state. This change of rebound properties involves no change in the connectivity. It only results from a switch between two types of excitability.

### A mean-field switch mechanism independent from connectivity properties

The novelty and significance of the switch mechanism at a network level is that it is largely independent of the network connectivity. Our paper differs in that regard from earlier computational studies that have studied modulations of network rhythms through modulations of the connectivity [39–41]. A common mechanism in those models is that stronger synchrony in the population relies on stronger connectivity [81]. In such models, *active* network states are associated to *weak* connectivity and asynchronous rhythmic activity whereas *oscillatory* states are associated to *strong* connectivity and synchronous rhythmic activity. Instead, in the present paper, the switch between the active and oscillatory state occurs without changes in connectivity. The connectivity is always weak, allowing for heterogeneous rhythms both in the active and oscillatory state. Stronger connectivity reduces the heterogeneity of rhythms in the population. Instead, a shared cellular switch allows for synchronous events even in the presence of heterogeneity.

### Network computational models often lack a cellular switch

Most network computational models in the literature lack the cellular switch studied in the present paper. This is evident for all models that focus on the synaptic connectivity and only model rate or spiking properties at a cellular level. But it is also the case for many models that account for bursting properties at a cellular level but lack a switch of excitability. Even models that include T-type calcium channels often model the activation as instantaneous [82–88]. Those models can simulate bursts or rebound properties for specific parameter values but the absence of a slow negative conductance makes them fragile and not tunable [42]. The fact that most models neglect the slow kinetics of the calcium channel activation provides further evidence of computational models that account for rebound mechanisms but do not account for a cellular switch of excitability. It illustrates that the distinction between the two mechanisms has not received much attention. This is not to say that modeling the switch requires more biophysical details than modeling the rebound. The recent paper [89] shows that a simple integrate-and-fire model is sufficient to model the switch provided that it contains distinct fast and slow thresholds to account for the two distinct types of excitability. It also explains why existing integrate-and-fire models, which have only one threshold, cannot model the excitability switch even in the presence of adaptation variables.

## Methods

All simulations were performed using the Julia programming language. Analysis were performed either in Julia or in Matlab. Julia and Matlab code files are freely available at http://www.montefiore.ulg.ac.be/~guilldrion/Files/Drionetal2018-Code.zip and *https://osf.io/k86en*.

**Fig 6:**
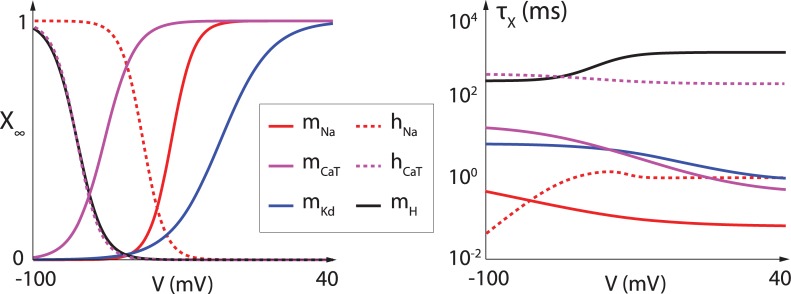
Steady-state channel gating curves (left) and time-constant curves (right) for the different ion channels present in the model neuron. Full lines represent activation curves. Dashed lines represent inactivation curves. Time-constants are shown on a log scale.

Single-compartment Hodgkin-Huxley models were used for all neuron models, following the equation $C_{m}\dot{V}=-\sum I_{ion}+I_{app}$, where *I*_*ion*_ corresponds to the ionic currents and *I*_*app*_ is an externally applied current. The model is composed of a leak current $I_{leak}={\bar{g}}_{leak}(V-V_{leak})$, a transient sodium current $I_{Na}={\bar{g}}_{Na}m_{Na}^{3}h_{Na}(V-V_{Na})$, a delayed-rectifier potassium current $I_{K,D}={\bar{g}}_{K,D}m_{K,D}^{4}(V-V_{K})$, a T-type calcium current $I_{Ca,T}={\bar{g}}_{Ca,T}m_{Ca,T}^{3}h_{Ca,T}(V-V_{Ca})$, a calcium-activated potassium current $I_{K,Ca}={\bar{g}}_{K,Ca}m_{K,Ca}([Ca])(V-V_{Ca})$ and a hyperpolarization-activated cation current $I_{H}={\bar{g}}_{H}m_{H}(V-V_{H})$, where *m* represents activation variables and *h* represents inactivation variables. The dynamics of voltage-gated activation and inactivation variables are modeled using the equation $\tau_{x}(V){\dot{m}}_{x}=m_{x,\infty }(V)-m_{x}$, where $m_{x,\infty }(V)=1/(1+\exp\left(\frac{V-V_{half}}{V_{slope}}\right))$ and $\tau_{x}(V)=A-B/(1+\exp\left(\frac{V-D}{E}\right))$, except for *τ*_*h*,*Na*_(*V*), which is given by *τ*_*hNa*_(*V*) = 0.67/(1 + exp((V + 62.9)/−10.0))) * (1.5 + 1/(1 + exp(V + 34.9)/3.6))). The values of *V*_*half*_, *V*_*slope*_, *A*, *B*, *D* et *E* for each variable are given in Table 1, and the corresponding curves are plotted in Fig 6. The calcium-dependent activation of the calcium-activated potassium current is modeled as follows: *m*_*K*,*Ca*_([*Ca*]) = ([*Ca*]/([*Ca*] + *K*_*D*_))^2^.

**Table 1:** Parameter values for steady-state channel gating curves and time-constant curves for the different ion channels present in the model neuron.

	*X*_∞_(*V*)		*τ*_*X*_(*V*)			
	*V*_*half*_	*V*_*slope*_	A	B	D	E
*m*_*Na*_	-35.5	-5.29	1.32	1.26	-120	-25
*h*_*Na*_	-48.9	5.18	/	/	/	/
*m*_*Kd*_	-12.3	-11.8	7.2	6.4	-28.3	-19.2
*m*_*CaT*_	-67.1	-7.2	21.7	21.3	-68.1	-20.5
*h*_*CaT*_	-80.1	5.5	410	179.6	-55	-16.9
*m*_*H*_	-80	6	272	-1149	-42.2	-8.73

Parameters used in simulations were as follows: *C*_*m*_ = 1 *μF*/*cm*^2^, *V*_*Na*_ = 50 *mV*, *V*_*K*_ = −85 *mV*, *V*_*Ca*_ = 120 *mV*, *V*_*leak*_ = −59 *mV*, *V*_*H*_ = −20 *mV*. All maximal conductance values were picked randomly with respect to a uniform distribution in the following ranges (in *mS*/*cm*^2^): ${\bar{g}}_{leak}\epsilon [0.0475,0.5575]$, ${\bar{g}}_{Na}\epsilon [135,205]$, ${\bar{g}}_{K,D}\epsilon [20,60]$, ${\bar{g}}_{Ca,T}\epsilon [0.375,0.725]$, ${\bar{g}}_{k,Ca}\epsilon [3,5]$, ${\bar{g}}_{H}\epsilon [0.0095,0.0105]$. Calcium dynamics followed the equation $\dot{\left[Ca\right]}=-k_{1}I_{Ca,T}-k_{2}[Ca]$ where *k*_1_ and *k*_2_ were also picked randomly with respect to a uniform distribution (*k*_1_ϵ [0.075,0.125] and *k*_2_ϵ [0.0075,0.0125]). In the case of instantaneous T-type calcium channel activation, the T-type calcium current was modeled as $I_{Ca,T}={\bar{g}}_{Ca,T}m_{Ca,T,\infty }^{3}(V)h_{Ca,T}(V-V_{Ca})$.

Neuron models were connected via AMPA, GABA_A_ and GABA_B_ connections using the following equations: $I_{AMPA}={\bar{g}}_{AMPA}AMPA(V-0),I_{GABA,A}={\bar{g}}_{GABA,A}GABAA(V-V_{Cl})$, and $I_{GABA,B}={\bar{g}}_{GABA,B}GABAB(V-V_{K})$, where *AMPA*, *GABAA* and *GABAB* are variables whose variation depends on the presynaptic potential *V*_*pre*_ following the equations $\dot{AMPA}=1.1\;T_{m}(V_{pre})[1-AMPA]-0.19AMPA$, $\dot{GABAA}=0.53\;T_{m}(V_{pre})[1-GABAA]-0.19GABAA$, $\dot{GABAB}=0.016\;T_{m}(V_{pre})[1-GABAB]-0.0047GABAB$, *T*_*m*_(*V*_*pre*_) = 1/(1 + *exp*(−(*V*_*pre*_ − 2)/5)). Synaptic weights were taken randomly with respect to a uniform distribution around a central value (${\bar{g}}_{syn}={\bar{g}}_{syn,central}\;\pm \;{\bar{g}}_{syn,central}/8$). AMPA receptor reversal potential was set to 0 *mV*, GABA_A_ receptor reversal potential was set to chloride reversal potential (*V*_*Cl*_ = −70 *mV*) and GABA_B_ receptor reversal potential was set to potassium reversal potential (*V*_*K*_ = −85 *mV*). GABA_B_ receptor activation was considered 50 time slower than GABA_A_ and AMPA receptor activation.

The local field potential (LFP) dynamics results from the collective synaptic activity of the neuronal population and is modeled by the normalized sum of the postsynaptic currents. The LFPs are low-pass filtered at 100 Hz via a fourth order Butterworth filter to reflect the use of macro-electrodes in LFP acquisition. The spectrogram analyses, or time-frequency plots, result from a logarithmic representation of the spectrogram of the short-time Fourier transform of the LFP. For the short-time Fourier transform, we consider a sampling frequency F_s_ = 1 kHz.

Spatial clustering of GABA_B_ connections was introduced by adding a Gaussian decay in the synaptic strength from neuron i to neuron j:

$$g_{SD}=g_{syn}e^{-\frac{{\left(j-i\right)}^{2}}{2c_{ij}^{2}}}$$

where *g*_*syn*_ is the maximal synaptic strength, *c*_*ij*_ is the space constant controlling the spread of connectivity (set to 0.8), and *i*,*j* are the positions of the neuron in the populations E and I. *g*_*SD*_ is normalized over the presynaptic population to get the same overall connection strength for each neuron in the postsynaptic population.

## Supporting information

S1 Fig Response of the model neuron with physiologically slow T-type calcium channel activation kinetics to the application of transient hyperpolarization for four different parameter sets. For the first parameter set (top left trace), a release of the hyperpolarization induces the generation of a transient spiking period in both models, a property called post-inhibitory rebound (PIR). For the second parameter set (top right trace), a release of the hyperpolarization induces the generation of a transient bursting period in the model having slow T-type calcium channel activation kinetics (left), a property called rebound bursting (RB). For the third and four parameter sets (bottom traces), a hyperpolarization induces a switch from slow tonic spiking to bursting, with the intraburst frequency being much higher than the tonic firing frequency, a property called hyperpolarization-induced bursting (HIB). The two different parameter sets both generate similar slow tonic firing frequency, a hyperpolarization-induced switch to bursting, but the bursting frequency is itself very different between the two, showing that the modulation of the rhythmic activity is independent from the switching mechanism.
(EPS)
